# Mesalazine as a cause of fetal anemia and hydrops fetalis: A case report: Erratum

**DOI:** 10.1097/MD.0000000000009623

**Published:** 2018-01-12

In the article, “Mesalazine as a cause of fetal anemia and hydrops fetalis: A case report”,^[1]^ which appeared in Volume 96, Issue 50 of *Medicine,* there are two typos. This sentence should appear as:

According to the SweidshMedical Birth Register 1302 pregnancies were reported exposed to sulfasalazine between 1973 and 2012, and 2072 to mesalazine, but these numbers may be underestimated.

The corresponding address should appear as Department of Laboratory Medicine, Karolinska Institutet, Karolinska University Hospital, Huddinge, 141 86 Stockholm, Sweden.
